# Electronic structure modulation for low-power switching

**DOI:** 10.1186/1556-276X-8-74

**Published:** 2013-02-13

**Authors:** Hassan Raza

**Affiliations:** 1 Department of Electrical and Computer Engineering, University of Iowa, Iowa City, IA, 52242, USA

**Keywords:** Electronic structure modulation, Graphene, Transistor, Electric field, Subthreshold slope, Gain

## Abstract

We report the transport characteristics of a novel transistor based on the electronic structure modulation of the channel. The gate voltage-controlled current modulation arises from the bandwidth manipulation of a midgap or a near-midgap state. We show that the transistor exhibits a gain and overcomes the 2.3 *k*_B_*T*/decade thermal limit in the inverse subthreshold slope where *k*_B_ is the Boltzmann constant. The unique device physics also opens up many novel applications.

## Background

We present a novel concept for modulating the channel transport by all-electronic means. The working principle is based on the electronic structure modulation of a midgap or a near-midgap state due to an electric field by applying a gate voltage. Small bandwidths (BW) have large effective masses and hence poor transport characteristics due to strong scattering. This leads to the off state of the transistor. The on state has a large bandwidth and hence smaller effective mass, which gives the higher desired conduction. The proposed transistor, namely electronic structure modulation transistor (EMT), has also been analyzed as a possible replacement for metal oxide semiconductor field-effect transistor technology [1].

Conventional field-effect transistors (FET) rely on the band edge shift using an external gate voltage. Hence, FETs are limited by the 2.3 *k*_B_*T*/decade thermal limit in their subthreshold inverse slope [2], where *k*_B_ is the Boltzmann constant and *T* is the temperature. With the scaling of the supply voltage, channel leakage current increases [2,3], making the power dissipation a serious challenge. It is, therefore, desirable to reduce the off current with a low supply voltage by overcoming the subthreshold thermal limit, while retaining the gain and high speed device (pico-second) and circuit (nano-second) operation. Various devices have been under study as possible candidates to replace FETs in complementary metal-oxide semiconductor (CMOS) technology [1].

Concepts based on the modulation of various device parameters have been explored earlier. For example, velocity/mobility modulation transistors rely on the real-space transfer of carriers between two adjacent materials with different mobilities [3]. Similarly, quantum modulation transistors are based on the constructive and destructive interference of the wavefunctions in the channel by electrically changing the T-shaped box dimensions [4]. Furthermore, quantum effects in various planar heterostructures based on the modulation-doped field-effect transistor principle have been explored [5], where the field-effect is used to perturb the barrier for carriers flowing between the source and the drain electrodes. The localization of the state near the band edges due to disorder in the Anderson localization is also a relevant concept, which leads to a mobility edge [6], but this effect is also limited by the thermal limit. In this paper, our objective is to show that the proposed EMT may become a viable candidate for low-power applications within the device and circuit performance constraints under extreme scaling.

## Methods

### Operating principle

**Figure 1:**
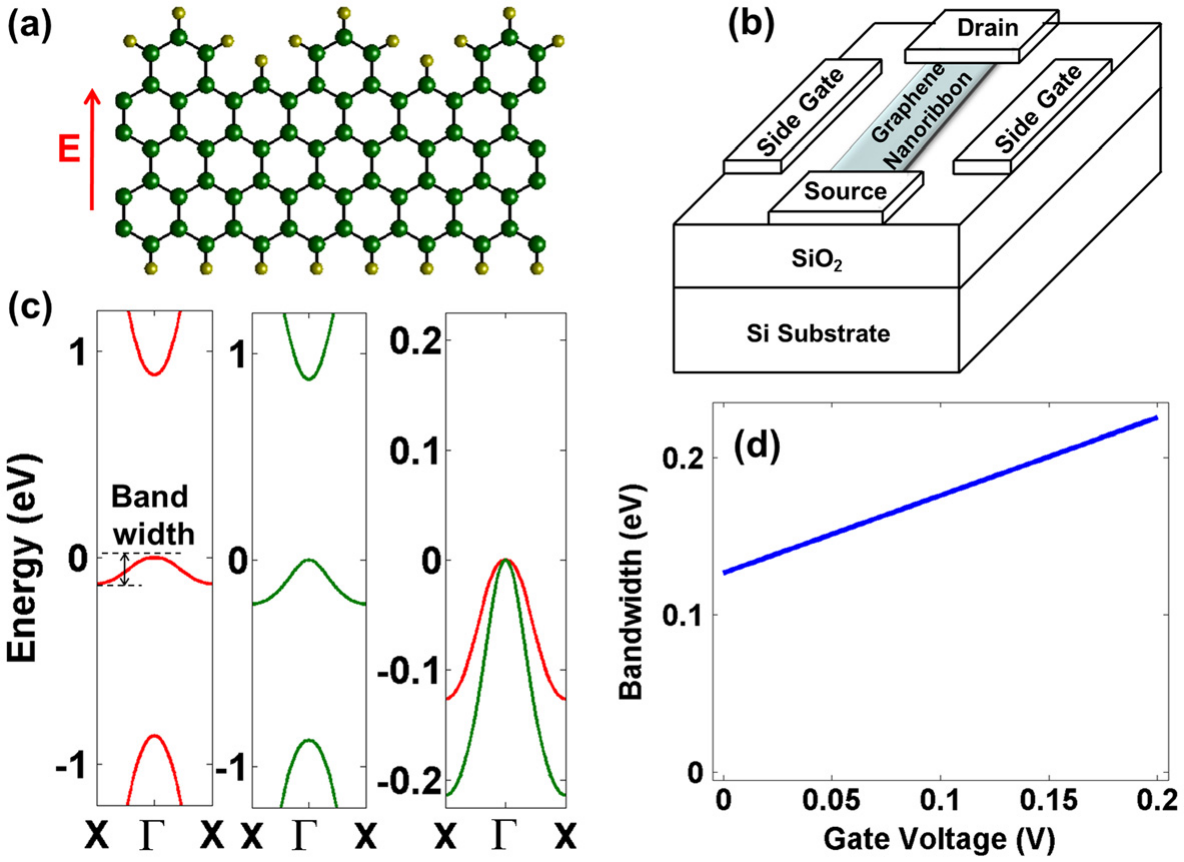
**Device structure and operating principle of an electronic structure modulation transistor.** (**a**) The channel consists of a 1-nm wide hydrogenated zigzag graphene nanoribbon with edge roughness. (**b**) The channel is side-gated to create an electric field in the width direction. Gate dielectric surrounds the channel and is not shown for clarity. (**c**) For such a ribbon, a near-midgap state with a small bandwidth is observed which can be modulated by the gate-induced electric field (left = 0 V/nm electric field, middle = 0.2 V/nm electric field, right = zoomed bandwidth comparison for the two electric fields). (**d**) The bandwidth of the near-midgap state is linearly dependent on the gate voltage [8].

A near-midgap state in the zigzag graphene nanoribbon (zzGNR) [7] with periodic edge roughness is extensively studied in [8]. In this work, we study novel device characteristics where the channel consists of a 1-nm wide zzGNR as shown in Figure 1a. The device structure is shown in Figure 1b, where the channel is gated by two side gates to create an electric field in the width direction. For such a side-gated nanoribbon, we show the electronic structure in Figure 1c using extended Hückel theory (see [8-12] for the detailed model). The two interesting electronic structure features are a significant band gap opening of about 2 eV, which is not very sensitive to the external electric field, and secondly a near-midgap state with a finite bandwidth, the bandwidth and dispersion of which can be manipulated by the gate-induced electric field. In Figure 1d, we show the dependence of the bandwidth on the gate voltage in the limit of relative permittivity of the gate dielectric to be much larger than that of the nanoribbon.

Such a bandwidth modulation can be understood in terms of the real-space localization of the wavefunction for various momentum values. At the *Γ* point, the wavefunction of the near-midgap state is distributed throughout the nanoribbon width, whereas at the *X* point is localized on the pristine edge. Additionally, the wavefunctions are localized on only one sublattice of graphene [8]. By applying a positive gate voltage at this edge, the energy values shift downward, thereby increasing the bandwidth as shown in Figure 1c. One should note that such modulation may happen due to intrinsic or extrinsic electric fields. In case of gate-voltage-induced modulation (extrinsic electric field) as shown in Figure 1d, the BW is given as follows:

(1) $$\mathrm{BW}=\mathrm{Mag}\left[\alpha \left|eV_{g}\right|+BW_{o}\right]\text{,}$$

where *α* is a dimensionless parameter, called the modulation factor. BW_o_ is the residual BW at zero gate voltage (Mag ≡ absolute magnitude) and *V*_g_ is the applied gate voltage. In Figure 1d, *α =* 0.47 and BW_o_ = 0.12 eV. With increasing width of the nanoribbon, the residual bandwidth BW_o_, the modulation factor *α*, and the band gaps are expected to decrease [8]. One could vary the device width, which will still result in qualitatively similar characteristics, as far as the conduction and valence band edges are well isolated from the near-midgap state.

Next, we consider the transport through the graphene nanoribbon by applying drain bias. In the limit of small drain bias, the channel transport is only dependent on the bandwidth of the near-midgap state. For zero bandwidth, no channel current flows through this state in the coherent limit, except for the dielectric leakage current and tunneling through the higher bands, which should be small given the conduction (valence) band is above (below) the localized state by about 1 eV. By applying a gate voltage to increase the bandwidth of the state, the channel current starts to flow. The operation of the EMT in this mode is equivalent to that of an *n*-MOS; hence, we refer to it as *n*-EMT. The equivalents of *p*-EMT can be realized by simply reversing the gate connections to induce an electric field in the reverse direction [8]. This all-electronic scheme thus operates under complementary mode. We envision that such transistor action is more general and can be achieved in any dimension with a near-midgap state in the channel region, the bandwidth of which can be modulated by the external voltage and for which, one can make ohmic contacts with the midgap state. In the limit of high bias, this transport picture changes, which we discuss later. So far, to the best of our knowledge, an experimental observation of such a state in a zzGNR has not been made.

### Theoretical model

To understand the transport in the high-bias regime, we consider a gedanken one-dimensional device and start with the ansatz of Equation 1. For such a device, we use single-band tight-binding approximation [13], where the channel bandwidth is 4|*t*_o_| and *t*_o_ is the nearest neighbor hopping parameter. For simplicity, we take five lattice points in the device region corresponding to a channel length and width of about 2 and 1 nm, respectively. The channel length can be decreased to about 1 nm as long as there is an unperturbed region in the middle with a near-midgap state, whereas the upper limit on the channel length can be bound by the scattering length, which can be in micrometer range for graphene. Similarly, the width can be varied as well which will result in a different gate voltage applied to achieve similar device characteristics. The Laplace’s potential due to the drain bias (*V*_d_) is included as a linear voltage drop. The Hartree potential is ignored for simplicity, since it does not affect the device operating principle, although it may affect the quantitative results. The choice of a simple model allows us to study the device and the circuit characteristics in terms of the modulation factor *α* and the residual bandwidth BW_o_. The transport characteristics of EMTs with different channel materials can thus be conveniently casted into this model very efficiently.

For the quantum transport, we use the non-equilibrium Green’s function formalism [14]. We consider the coherent limit where it is equivalent to the Landaüer’s approach, and the current can be evaluated from the transmission as below:

(2) $$I_{d}=2\left(\mathrm{forspin}\right)\frac{q}{h}\int \;\mathrm{dET}\left(E\right)\left[f_{s}-f_{d}\right]\text{,}$$

where transmission is *T*(*E*) = *tr*(*Γ*_s_*GΓ*_d_*G*^+^). The Green’s function for the channel is

(3) $$G={\left[\left(E+i0^{+}\right)I-H-U_{L}-\Sigma_{s,d}\right]}^{-1}\text{,}$$

where *I* is an identity matrix and *U*_L_ is the Laplace’s potential drop. Self-energies and broadening functions are $\Sigma_{s,d}=-t_{o}e^{ik_{s,d}a}$ and *Γ*_s,d_ = *i*[*Σ*_s,d_ − *Σ*_s,d_^+^], respectively. $f_{s,d}={\left[1+e^{\left(E-\mu_{s,d}\right)/k_{B}T}\right]}^{-1}$ are the contact Fermi functions. *μ*_s,d_ are source/drain chemical potentials. *μ*_d_ is shifted due to drain bias as *μ*_d_ = *μ*_o_ − *qV*_d_ and *μ*_s_ = *μ*_o_, where *μ*_o_ is the equilibrium chemical potential.

## Results and discussion

**Figure 2:**
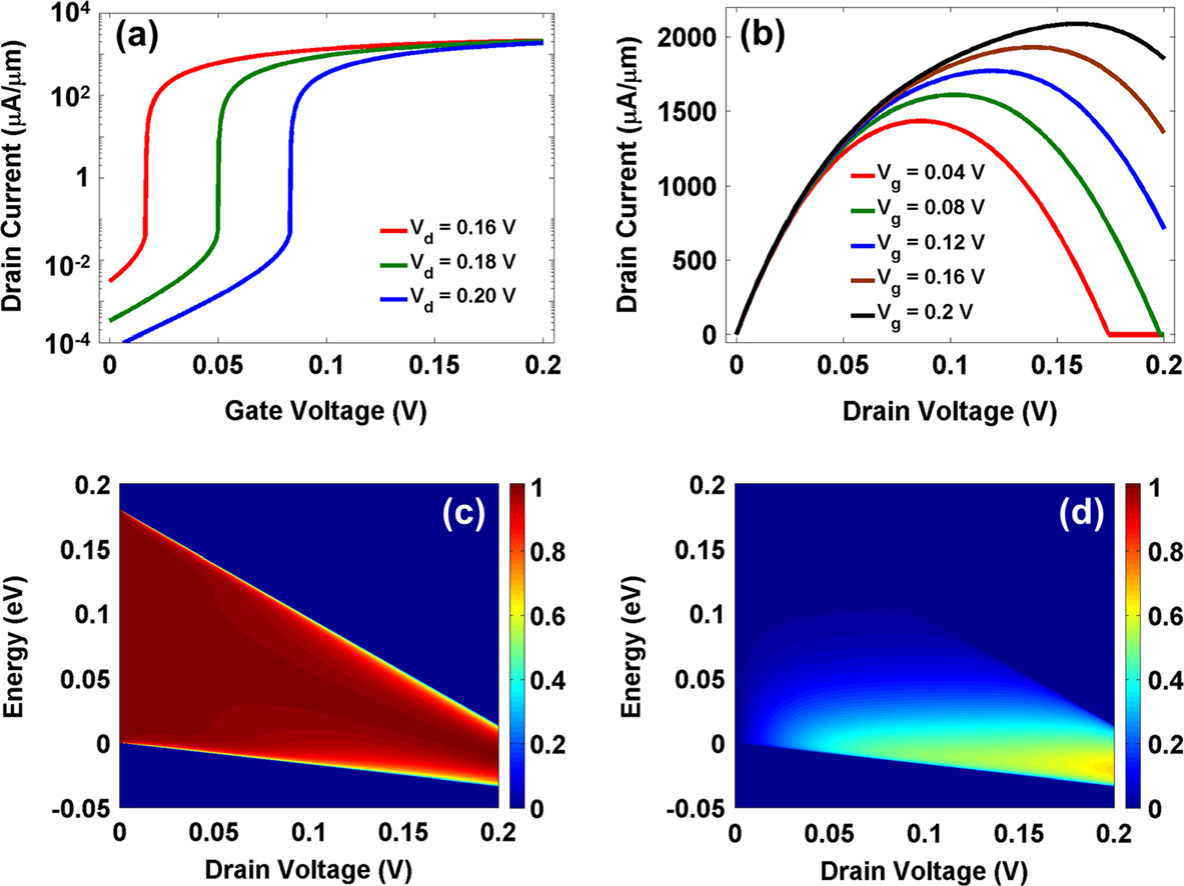
**Transport characteristics.** (**a**) Transfer characteristics show steep subthreshold characteristics with drain-voltage dependent threshold voltage shift. (**b**) Output characteristics show a saturating behavior followed by a negative differential resistance. (**c**) With increasing drain bias, the transmission window shrinks due to a spectral misalignment (Addition file 1). (**d**) The increasing Fermi function difference between the two contacts and the decreasing transmission lead to an increasing and then decreasing *T*(*E*)[*f*_s_ − *f*_d_] function.

We next discuss the numerical results for a transistor with *α* = 0.4 and BW_o_ = 0.1 eV. The transfer characteristics with *V*_d_ = 0.16, 0.18 and 0.2 V are shown in Figure 2a. A steep subthreshold slope is obtained with a high on/off current ratio. The threshold voltage depends on the drain voltage *V*_d_, and it increases with the drain bias - a trend opposite to the drain-induced barrier lowering of a FET. The subthreshold current much below the threshold voltage, which is due to the reflections from the barrier of the near-midgap state, decreases exponentially.

We further report the output characteristics in Figure 2b for *V*_g_ = 0.04, 0.08, 0.12, 0.16, and 0.2 V, which show a negative differential resistance (NDR) behavior that is crucial for the low-power inverter operation (Additional file 1). The current cut-off mechanism is similar to the Bloch condition through minibands in superlattices, giving rise to an NDR event, when the drain voltage exceeds the miniband width [15,16]. The miniband in superlattices is formed by the overlap of quantized states through tunnel barriers, inherently leading to small miniband widths and large effective masses [17]. The NDR events mediated by minibands have been reported in III-V heterostructures [18] and graphene superlattices [19]. However, the peak-to-valley ratio in such structures is limited to about 1.1 to 1.2. In comparison, the NDR feature reported for near-midgap state in this work shows a peak-to-valley ratio of greater than 10^3^, which is important for the low-power operation. The reason behind a higher ratio could be the formation of a midgap state band without any tunnel barriers, giving rise to higher conduction to yield a large peak current compared to the role of tunnel barriers in the miniband conduction, which lead to a small peak current due to quantum mechanical tunneling. On the other hand, the near-midgap state in this work is highly sensitive to the edge geometry. Therefore, achieving high material quality (with defect density less than parts per billion) is imperative for a proper operation of the proposed transistor. Moreover, the bandwidth of the near-midgap state is gate-voltage dependent; the *V*_d_ corresponding to peak and valley currents increases with increasing gate bias *V*_g_ due to a larger conduction window. Such peculiar drain voltage-dependent transport features are not exclusive for this device. In a three-terminal device, the electrostatics due to the drain bias introduces various non-trivial effects, e.g., pinch-off in FETs, etc.

To understand these device characteristics further, we report the drain bias dependence of the transmission window in Figure 2c for a gate voltage of 0.2 V. Without any drain bias, a wide transmission window is observed, which monotonically decreases with increasing bias (see Additional file 1 for further discussion). It is more interesting to look at the product of the transmission and the Fermi function difference of source/drain contacts *T*(*E*)[*f*_s_ − *f*_d_]. With the increasing bias, since the Fermi function difference monotonically increases, the overall trend as shown in Figure 2d is observed. Using Equation 2, one can also relate these trends in Figure 2d to the negative differential resistance trends of Figure 2b.

In the reported device, the threshold voltage can be engineered by optimizing the side gate electrostatics to vary the modulation factor *α*. Yet, another way to change the threshold voltage is by engineering the work function of the side gate materials to create an intrinsic electric field, thereby changing the BW_o_. *n*-EMT device characteristics are shown in Figure 2. Similarly, by gate work-function and dielectric engineering, one can also achieve *p*-EMT characteristics by reversing the gate connections. Moreover, the optical phonon energy in graphene is about 200 meV. The choice of 0.2 V supply voltage allows us to ignore the electron–phonon inelastic scattering in these calculations.

**Figure 3:**
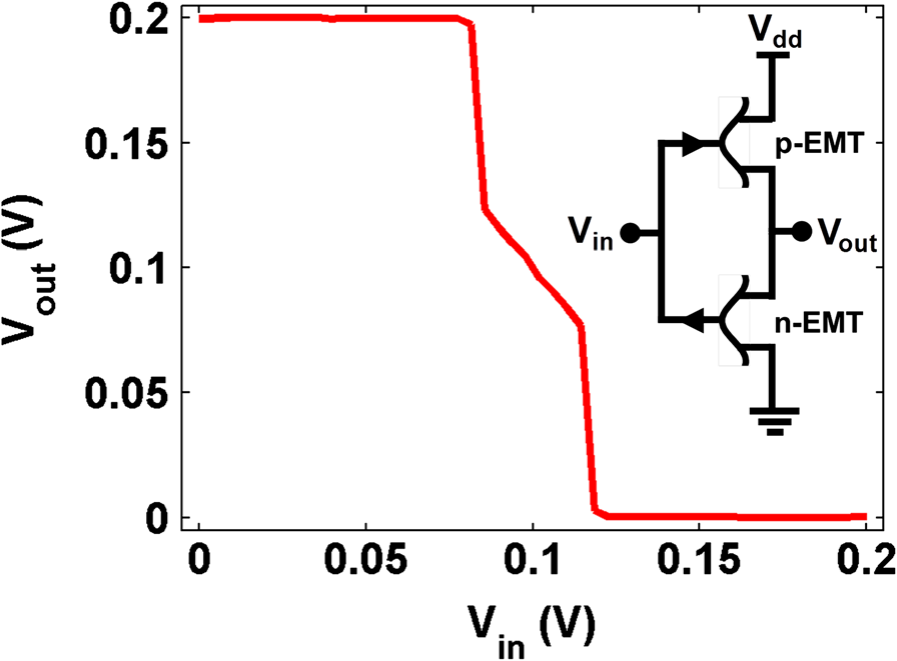
**Inverter characteristics.** EMT inverter shows a large gain and appreciable noise margins. The circuit diagram with *p*- and *n*-EMTs is shown in the inset.

Next, we calculate the inverter characteristics using the complementary characteristics in Additional file 1. The voltage transfer curve of an inverter, formed by a *p*-EMT and an *n*-EMT connected back to back, is shown in Figure 3. The proposed symbols for *n*-EMT and *p*-EMT are also shown. The transfer characteristics show a steep slope. The high and low noise margins are 0.082V, which ensure a self-correcting digital operation. The maximum magnitude of gain is about 18, whereas the magnitude of gain around 0.1 V of input/output voltage is about 1.6. Although EMT has a severe drain bias-dependent threshold voltage shift, the NDR in the output characteristics ensures that the power dissipation occurs only during the switching (see Additional file 1 for further discussion). Moreover, the stable state around 0.1 V input voltage becomes more interesting, which can be used to build three-valued logic and memory devices.

## Conclusions

We have reported an all-electronic transistor with low supply voltage based on the electronic structure modulation of a near-midgap state in the channel using an external gate voltage. The device physics, however, may lead to various applications of technological importance. We have shown that one can obtain gain and large on/off channel current ratio with few *k*_B_*T* supply voltage. We envision that the transistors based on the electronic structure modulation can open up a new class of post-CMOS logic devices. The concept is analyzed in zzGNR, provided the challenges related to the atomic control of the graphene nanoribbon edge quality and side gate electrostatics, and ohmic contacts with the near-midgap state can be overcome.

## Competing interests

Author declares that he has no competing interests.

## Authors’ information

HR is an assistant professor in Electrical and Computer Engineering at the University of Iowa since May 2009. For two years, he was a postdoctoral associate at Cornell University. He received his BS on July 2001 from the University of Engineering and Technology Lahore Pakistan, MSc on December 2002, and Ph.D. on May 2007 from Purdue University. He has received “Magoon Award for Excellence in Teaching” from Purdue University. He is also the recipient of “Presidential Faculty Fellowship” and “Old Gold Fellowship” from the University of Iowa. His research group is focused on “anything that is small” for low-power post-CMOS transistor, spintronics, sensors, and solid-state energy harvesting applications from theoretical, experimental, and computational approaches using graphene, molecule, silicon, novel dielectrics, and carbon nanotube material systems. He has served as an editor of a 600-page book on Graphene Nanoelectronics published by Springer in 2012.

## Supplementary Material

Additional file 1 **Supplementary information.** Channel conduction window and output characteristics for *n-*EMT. Click here for file
