# Supplementary material for: Electronic structure modulation for low-power switching
Source: Nanoscale Res Lett. 2013 Feb 13;8(1):74. doi: 10.1186/1556-276X-8-74 (PMC3606601; doi:10.1186/1556-276X-8-74)
Supplement: Additional file 1 — Supplementary information. Channel conduction window and output characteristics for n-EMT. [file 1556-276X-8-74-S1.docx]

**Supplementary Information:**

Figure A: Channel conduction window - Case I: small bandwidth (due to small gate voltage) does not result in a conduction window between source and drain; Case II: even a slightly larger bandwidth is not sufficient; Case III: a conduction window is achieved for a larger bandwidth; Case IV: a further increase in drain bias results in a small conduction window for the same bandwidth as in case III.

The Laplace potential due to the drain bias can block the conduction under coherent conditions and delay the onset of conduction schematically explained in Fig. A. Apart from the dielectric leakage current, zero BW results in a zero channel current. However, an incremental gate voltage, only slightly increasing the BW, would not lead to any transport because the drain bias also shifts the energy windows linearly for various lattice points. A conduction window thus cannot be established as shown for cases I and II. Substantial gate voltage is required to make a conduction window between source and drain as shown in case III. In other words, the threshold voltage depends on the drain bias.

In coherent transport, the lower limit of transmission as a function of the drain bias is due to the lower band edge shift of the lattice point closer to the source. Similarly the upper limit is due to the upper band edge shift of the lattice point closer to the drain as shown in Fig. A. A higher drain bias reduces this conduction window as schematically shown in case IV, which leads to a negative differential resistance.

Figure B: Output characteristics for n-EMT (shown by black dashed line) and p-EMT (shown by red solid line) for inverter operation. Arrows show the direction of increasing input voltage (V_in_).

Output characteristics at various input voltages (V_in_) ranging from 0 to 0.2V are shown in Fig. B. For a given input voltage, the intersection of n-EMT and p-EMT current-voltage characteristics give the inverter operating point. One observes that despite severe drain bias dependent threshold voltage shift in the transfer characteristics, the NDR (negative differential resistance) in the output characteristics is the key characteristic to achieve low-power inverter operation.
